# High NEK2 confers to poor prognosis and contributes to cisplatin‐based chemotherapy resistance in nasopharyngeal carcinoma

**DOI:** 10.1002/jcb.27632

**Published:** 2018-10-08

**Authors:** He Xu, Liang Zeng, Yongjun Guan, Xiangling Feng, Yinghong Zhu, Yichen Lu, Chen Shi, Shilian Chen, Jiliang Xia, Jiaojiao Guo, Chunmei Kuang, Wei Li, Fengyan Jin, Wen Zhou

**Affiliations:** ^1^ Cancer Center, The First Hospital of Jilin University Changchun China; ^2^ Cancer Research Institute, Central South University; Key Laboratory of Carcinogenesis and Cancer Invasion, Ministry of Education; Key Laboratory of Carcinogenesis, National Health and Family Planning Commission Changsha Hunan China; ^3^ Hunan Cancer Hospital and the Affiliated Cancer Hospital of Xiangya School of Medicine, Central South University Changsha Hunan China; ^4^ School of Public Health, Central South University Changsha Hunan China

**Keywords:** cisplatin‐based chemotherapy resistance, nasopharyngeal carcinoma (NPC), NEK2

## Abstract

Nasopharyngeal carcinoma (NPC) is a common malignant tumor in southern China and Southeast Asia, but the molecular mechanism of its pathogenesis is poorly understood. Our previous work demonstrated that NEK2 is overexpressed in multiple cancers. However, how NEK2 involves in NPC development remains to be elucidated. In this study, we firstly identified NEK2, located at +1q32‐q33, a late event in NPC pathogenesis, overexpressed in the stage III‐IV and paired sequential recurrent patients with NPC by immunohistochemistry. Furthermore, Kaplan‐Meier analysis indicated high NEK2 conferred an inferior overall survival in NPC. In addition, cisplatin experiments with cell counting kit‐8, colony formation, and a xenograft mice model of NPC demonstrated that NEK2 contributed to proliferation and cisplatin resistance in vitro and in vivo. On the contrary, downregulation of NEK2 by short hairpin RNA inhibited NPC cell growth and increased the sensitivity of cisplatin treatment in vitro. Thus, increased expression of NEK2 protein could not be predicted for poor survival but used as a novel biomarker for recurrence of NPC. Targeting NEK2 has the potential to eradicate the cisplatin‐based chemotherapy resistant NPC cells.

## 1 INTRODUCTION

Nasopharyngeal carcinoma (NPC) is a malignant tumor that occurs in nasopharyngeal mucosa and belongs to head and neck cancer. It has high incidence in Southern China.^1^ Currently, the standard treatment for NPC is radiotherapy and concurrent/adjuvant chemotherapy, using drugs such as cisplatin and related cisplatin‐base compounds. Although the combined therapeutic regimen has greatly improved the complete response at diagnosis and prolonged the overall survival at 5 years from 56% to 62%,^2^ a number of patients suffer from therapeutic resistance, local recurrence, and distant metastases. The major reasons for cancer treatment failure are the unclear mechanism of NPC pathogenesis and the existence of drug‐resistant subclones developed during treatment. Several groups have identified several potential biomarkers, including NFBD1, COX2,^3^ LMP‐1^4^, and MMP19 etc, for predicting NPC recurrence and radio‐chemotherapy resistance.^5^ However, the mechanisms of NPC progression and recurrence remain unclear. Therefore, there is an urgent need to explore novel related markers to serve as diagnostic markers and molecular targets for NPC.

Previously, we identified a critical protein, NEK2, one of the never in mitosis associate (NIMA) kinase family – which has several putative roles in cell division, most notably in spindle formation and chromosome segregation^6^ – to be highly associated with poor prognosis and inferior survival in several different cancers, including head and neck squamous cell carcinoma, bladder carcinoma, glioblastoma, T‐cell acute lymphoblastic leukemia, colon carcinoma, hepatocellular carcinoma, melanoma, and ovarian adenocarcinoma, by the gene expression profiling (GEP) analysis. Furthermore, we confirmed that both PP1/AKT and Wnt pathways are involved in NEK2‐induced cancer cell drug resistance, proliferation, and chromosomal instability in multiple myeloma and lung cancer. Meanwhile, several groups have also demonstrated elevated levels of NEK2 protein expression through E2F4 induced tumorigenesis,^7^ upregulation of NEK2 by microRNA‐128 methylation associated with a poor prognosis in colorectal cancer,^8^ and NEK2 expression is upregulated in cisplatin drug‐resistant ovarian cancer cells.^9^ Considering that NPC is a malignant genomic instability disease with ‐3p26–13 (48.9%), ‐11q22 to 25 (38.1%), ‐16q12 to 24 (38.1%), ‐14q24 to 32 (32.4%), ‐13q21 to 32(22.3%), ‐9p23–21(21.6%), +12p12 (46%), +12q13 to 15 (43.9%), +1q22 to 32 (33.1%), +3q13.1 to 26.2 (30.2%), and +8q22.1 to 24.2 (27.3%)^10^ and that NEK2 is involved in the chromosome segregation and also located in the +1q22 to 32, we proposed that NEK2 might be involved in NPC progression.

Our goal in this study is to identify NEK2 as a reliable, clinically useful prognostic marker through examining the effects of NEK2 overexpression on disease progression and drug resistance and exploring how high expression of NEK2 induces drug resistance in the NPC cells.

## 2 MATERIALS AND METHODS

### 2.1 Cell culture

The nasopharyngeal carcinoma cells (CNE2 and CNE2DDP, a cisplatin resistance cell line) were cultured in Roswell Park Memorial Institute (RPMI) 1640 medium with 10% fetal calf serum, 100 IU/mL penicillin, and 100 mg/mL streptomycin at 37°C in a humidified atmosphere of 5% CO_2_.

### 2.2 Patients samples

Eight chronic nasopharyngitis (NP) biopsies and 23 primary poorly‐differentiated NPC biopsies were obtained with consent before treatment at the Xiangya Hospital of Central South University (CSU) in 2016 and 2017. A total of 173 paraffin‐embedded specimens, including 26 NP samples, 118 NPC samples, and 29 paired (at diagnosis and recurrence) sequential samples, were supplied by Hunan Tumor Hospital (Changsha, Hunan, China), CSU from 2002 to 2012. The histological patterns and clinical stages of NPC were classified as follows: 80 cases of male and 38 cases of female, 73 cases of age less than and equal to 50 years old, 45 cases of age more than 50 years, 75 cases of T1 to 2, and 43 cases of T3 to 4. Among the patients included in the research, 91 were positive for cervical lymph node metastasis (N1/N2/N3 = 91) and 27 were negative (N0 = 27); there were 7 cases of distant metastasis and 111 cases of no metastasis; 35 cases of clinical stage I‐II; and 83 cases of stage III‐IV. Among 118 patients, 86 patients by RT alone, and 32 patients by CCRT. Complete clinical record and follow‐up data of all patients were available. The overall survival time was calculated from the data of diagnosis to the date of death or the data last known alive. A total of 63 patients (53.4%) were alive and 55 patients (46.6%) were dead with a mean follow‐up period of 58.2 months (2‐98 months) (Table 1). All the specimens were stained with hematoxylin and eosin (HE) for histological examination and reviewed by an otorhinolaryngologic pathologist. The present study was approved ethically by the cancer research institute review board of CSU.

**Table 1:** The clinicopathologic parameters of 118 patients with NPC

Variable	No. of patients	%
Sex		
Male (n = 80)	80	67.80
Female (n = 38)	38	32.20
Age		
≦ 50 y (n = 73)	73	61.86
> 50 y (n = 45)	45	38.14
Tumor size		
T1‐2 (n = 75)	75	63.56
T3‐4, (n = 43)	43	36.44
Lymph node status		
N = 0 (n = 27)	27	22.88
N = 1,2,3 (n = 91)	91	77.12
Distant metastasis		
M = 0 (n = 111)	111	94.07
M = 1 (n = 7)	7	5.93
Clinical stages		
StageI‐II (n = 35)	35	29.66
StageIII‐IV (n = 83)	83	70.03
Survival status		
Alive (n = 63)	63	53.39
Death (n = 55)	55	46.61

Abbreviation: NPC, nasopharyngeal carcinoma

### 2.3 Immunohistochemistry staining and statistical analysis

Immunohistochemistry (IHC) staining was used to analyze the expression of NEK2 protein in NP and NPC tissues including different stages and paired (at diagnosis and recurrence) sequential samples, according to the protocol described in our previously published paper.^11^ Briefly, the sections were incubated in H_2_O_2_ (3%) for 10 minutes, blocked in 1% bovine serum albumin for 60 minutes and then incubated with NEK2 antibody overnight at 4°C (NEK2 rabbit antibody; Cat#. SC33167; Santa Cruz Biotechnology, CA), followed by secondary antibody incubation (Cat#. kit9922; MXB Biotechnologies, FuZhou, China) for 60 minutes. Then, staining was performed with the view 3,3'‐diaminobenzidine staining fluid (DAB) detection kit (Cat#. DAB‐0031; MXB Biotechnologies, China). Sections were then counterstained with hematoxylin, dehydrated, and mounted. The staining was observed under a microscope. Semiquantitative assessment of NEK2 immunostaining was performed by calculating both intensity of staining (0, 1, 2, or 3) and extent of staining (0, 0%; 1, <10%; 2, 10‐50%; 3, >50%). The scores for the intensity of staining and extent of staining were multiplied to give a weighted NEK2 score for each case (maximum possible, six). All immunohistochemical staining was evaluated and scored by at least two independent pathologists.

### 2.4 Plasmids and virus production

NEK2 cDNA clones were purchased from Invitrogen and cloned into the pCDH‐CMV‐MCS‐EF1‐copGFP vector. The target short hairpin RNA (shRNA) for NEK2 sequence 5′‐GAT CCC CGG AGG AAG AGT GAT GGC AAG ATT CAA GAG ATC TTG CCA TCA CTC TTC CTC CTT TTT A‐3′ was obtained from Invitrogen as we previously performed, NEK2 shRNA double‐stranded oligonucleotides were cloned into pLenti‐CMV‐EGFP‐MCS. Recombinant lentivirus was produced by transient transfection of 293T cells. After lentivirus transduction, NEK2 overexpressed NPC cells were purified by flow cytometry sorting, and NPC cells expressing NEK2 shRNA were selected with puromycin (2.5 g/mL).

### 2.5 Real‐time reverse transcription‐polymerase chain reaction and Western blot analysis

Real‐time reverse transcription‐polymerase chain reaction (RT‐PCR) analysis was performed as previously described.^12^ Briefly, total RNAs were extracted using TRIzol reagent (Cat#.15596026; Invitrogen, Life Technology, MA), and complementary DNAs (cDNA) were synthesized using Revert Aid First Strand cDNA Synthesis Kit (Cat#. K1622; Thermo Fisher Scientific, MA) according to the manufacturer's instructions. Real‐time quantitative PCRs (qRT‐PCR) for NEK2 and GAPDH were performed with AceQ qPCR SYBR Green Master Mix (Cat#. Q111‐02; Vazyme Biotechnology, Shanghai, China) on the CFX connect real‐time system (Bio‐Rad). The mRNA levels of NEK2 relative to glyceraldehyde phosphate dehydrogenase (GAPDH) were calculated using the ${▵▵C}_{t}$ method. Western blot analysis was performed as described previously.^13^ Briefly, total proteins were extracted with radio immunoprecipitation assay (RAPI) strong buffer (Cat#. p0020; Auragene Bioscience, Changsha, China), and protein concentrations were measured using a bicinchoninic acid (BCA) protein Assay Kit (Cat#. BCA‐02; BeiJing Dingguo Biotechnology, Beijing, China). Proteins were then separated by 10% sodium dodecyl sulfate (SDS)‐polyacrylamide gel electrophoresis and transferred to 0.45‐μm polyvinylidene fluoride (PVDF membranes; Cat#.IPFL00010; Millipore). The blots were then probed with specific primary antibodies (NEK2 1:1000; SC33167; Santa cruz; GAPDH 1:5000; Cat#. SC25778; Santa Cruz, CA) overnight at 4°C, followed by HRP‐conjugated secondary antibody (goat anti‐rabbit; Cat#. SC2004; Santa Cruz, CA) incubation for 2 hours at room temperature. Protein signals were developed with Millipore LuminataTM Crescendo Western HRP Substrate (Cat#. WBLUC 0500; Millipore, MA). The developed images were obtained and analyzed using ChemiDox XRS Chemiluminescence imaging system (Bio‐Rad).

### 2.6 Colony formation assay and growth curve

Colony formation assay was performed as previously described.^14^ Briefly, NPC cells (1000 cells/well) were plated in six‐well plates, and the next day, cells were exposed to the indicated treatment. After 10 to 12 days, the cells were fixed and stained with 0.1% crystal violet. One colony was defined if more than 50 cells were observed. Colonies were imaged and enumerated using the Image J software. Cell growth was examined at different time points (0, 24, 48, and 72 hours) through cell counting‐8 kit (CCK‐8) according to the manufacturer's instructions (CCK‐8, Cat#.B34304; Biotool, Selleck Biotechnology, Shanghai, China).

### 2.7 Flow cytometry for apoptosis

Cell apoptosis were measured by PE‐annexin V apoptosis detection Kit (Cat#. 559763; BD, NewYork) according to the manufacturer's instructions. The cells (1 × 10^5^) were washed in PBS and suspended in 1 mL binding buffer. PE‐conjugated antibody to annexin V (5 μL), and 7‐AAD (5 μL) were added to the cell suspension. Cells were incubated for 30 minutes at 37°C, washed and re‐suspended in binding buffer and subjected to flow analysis. The results were analyzed using the FlowJo software.

### 2.8 Xenograft model in nude mice

All animals' works were performed in accordance with the guidelines of the Institutional Animal Care and local veterinary office and ethics committee of the CSU, China (animal experimental license no 2017sydw0289) under approved protocol. CNE2 cells with pCDH vector or pCDH NEK2 OE (8 × 10^5^ cells in 100 µL PBS) were injected subcutaneously into the left armpit of NOD mice. Tumor burdens were monitored by measuring tumor volumes every 3 days. Tumor volumes were calculated according to the equation V = (length × width^2^)/2.

### 2.9 Gene expression profiling analysis

GEP and clinical data were derived from NIH Gene Expression Omnibus (http://www.ncbi.nlm.nih.gov/geo/). Two GEP data were used in this study, including GDS3341 and GDS3610.

### 2.10 Statistical analysis

Data were presented as mean ± standard error. An analysis of variance and *t* test were used for statistical analysis. A value of *P* ≤ 0.05 was considered to be significant.

## 3 RESULTS

### 3.1 High NEK2 confers to poor survival in patients with NPC

**Figure 1:**
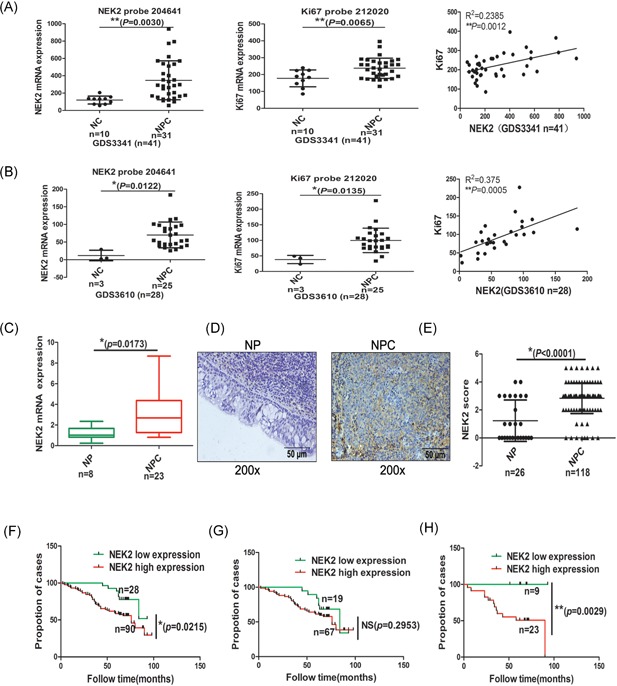
High NEK2 confers to poor survival in patients with NPC. A and B, GEP analysis of NEK2 and Ki67 in NPC and normal nasopharyngeal epithelia derived from two public GEP databases (GDS3341 and GDS3610, respectively, *n* = 41 and *n* = 28). C, real‐time PCR was performed to detect NEK2 mRNA expression in NP (*n* = 8) and NPC (*n* = 23). D and E, NEK2 protein expression was detected in NP (*n* = 26) and NPC (*n* = 118) by IHC. F‐G‐H, Kaplan‐Meier analysis overall survival was performed on patients with NPC, treated with radiotherapy (RT) or concurrent chemoradiotherapy (CCRT) according to NEK2 expression level. GEP, gene expression profiling; IHC, immunohistochemistry; mRNA, messenger RNA; NP, nasopharyngitis; NPC, nasopharyngeal carcinoma; PCR, polymerase chain reaction

Because we reported that NEK2 was highly expressed in the head and neck carcinoma, we further compared two public GEP datasets for NPC and normal nasopharyngeal epithelia control (NC) including 41 (10 NC and 31 NPC) and 28 (3 NC and 25 NPC) cases, respectively. In these two GEP database, NEK2 were over expressed in NPC compared with NC (*P* = 0.0030 and *P* = 0.0122). We analyzed the expression of several proliferation‐related genes in GEP datasets. Among which, Ki67, a cell proliferation marker, was found to be significantly increased in NPC compared with normal (*P* = 0.0065 in GDS3341; *P* = 0.0135 in GDS3610); moreover, Ki67 showed a positive correlation with NEK2 in NPC (*R* = 0.2385, *P* = 0.0012 in GDS3341; *R* = 0.375, *P* = 0.0005 in GDSD3610) (Figure 1A and 1B). To further validate the GEP results, we collected 23 specimens of nasopharyngeal carcinoma and eight specimens of nasopharyngitis to examine the mRNA expression by RT‐PCR. The results showed that NEK2 was also higher expressed in patients with NPC significantly (*P* = 0.0173) (Figure 1C).

To examine the clinical significance of high NEK2 expression in NPC, we examined the NEK2 protein expression in paraffin sections including 26 NP and 118 NPC. As shown in Figure 1D, there was significantly higher expression of NEK2 protein, mainly expressed in cytoplasm in NPC compared with NP, indicating that upregulation of NEK2 is involved in NPC pathogenesis (*P* < 0.0001, Figure 1E).

We further analyzed the associations between protein expression of NEK2 and the clinical pathological features including gender, age, clinical stages, tumor size, lymph node status, survival status, distant metastasis, and treatment by the Chi‐square test. The results showed a strong positive correlation between NEK2 expression and clinical stages of NPC, and the advanced clinical stages (stage III‐IV) had a significantly higher positive percentage of NEK2 expression than that in the early stages of NPC (*P* = 0.034) (Table 2). To further examine the impact of NEK2 expression on the survival status of patients with NPC, Kaplan‐Meier analysis was applied to analyze the survival curve of all 118 patients with NPC. Figure 1F illustrates that patients with high expression of NEK2 have poor survival than low expressed (*P* = 0.0215). Furthermore, we further analyzed the correlation between NEK2 expression and treatment. There was no significant difference between NEK2 high expressed patients and low expressed patients treated with RT alone (Figure 1G), while the high expression of NEK2 conferred inferior survival among patients with NPC treated with CCRT (*P* = 0.0029) (Figure 1H). Again, the effect of NEK2 on gender, age, lymph node status, and distant metastasis was not detected (Table 2 and 3).

**Table 2:** Analysis of the association between NEK2 protein expression and clinicopathological features of NPC

Clinicopathological features, n	NEK2 expression		
	Negative, %, score ≦ 2	Positive, %, score > 2	*P*‐value
Sex			
Male (n = 80)	19(24)	61(76)	1.000
Female (n = 38)	9(24)	29(76)	
Age			
≦ 50 y (n = 73)	18(25)	55(75)	0.827
> 50 y (n = 45)	10(22)	35(78)	
Tumor size			
T1‐2 (n = 75)	20(27)	55(73)	0.374
T3‐4 (n = 43)	8(19)	35(81)	
Lymph node status			
N = 0 (n = 27)	8(30)	19(70)	0.290
N = 1,2,3 (n = 91)	20(22)	71(78)	
Distant metastasis			
M = 0 (n = 111)	26(23)	85(77)	0.669
M = 1 (n = 7)	2(29)	5(71)	
Clinical stages			
StageI‐II (n = 35)	13(37)	22(63)	0.034^*^
StageIII‐IV (n = 83)	15(18)	68(82)	
Survival status			
Alive (n = 63)	20(32)	43(68)	0.032^*^
Death (n = 55)	8(15)	47(85)	

Abbreviation: NPC, nasopharyngeal carcinoma

^*^ The Chi‐square test, statistically significant difference (*P* < 0.05).

**Table 3:** Analysis of the association between NEK2 protein expression and survival status in different therapeutic strategy

	NEK2 expression		
Therapeutic strategy	Negative (score ≦ 2)	Positive (score > 2)	*P* value
Radiotherapy (RT), *n* = 86			
Alive (*n* = 43)	11	32	*P* = 0.604
Death (*n* = 43)	8	35	
Chemotherapy combined with radiotherapy (CCRT), *n* = 32			
Alive (*n* = 21)	9	12	*P* = 0.013^*^
Death (*n* = 11)	0	11	

^*^ The Chi‐square test, statistically significant difference (*P* < 0.05).

### 3.2 Over‐expressed NEK2 contributes to proliferation in vitro and in vivo

**Figure 2:**
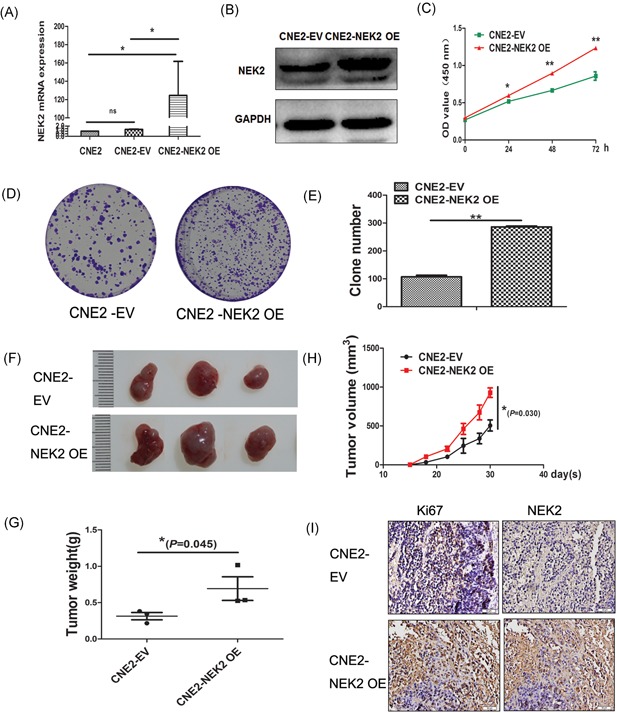
Overexpression of NEK2 contributes to proliferation in vitro and in vivo. A, analysis of NEK2 mRNA expression in CNE2‐NEK2 OE cells. B, NEK2 protein expression was detected in CNE2‐NEK2 OE cells. C, the proliferations of CNE2‐EV and CNE2‐NEK2 OE were detected by CCK‐8 at different time points. D and E, the colony formations of CNE2‐EV and CNE2‐NEK2 OE were observed at day 10. Results were expressed as means ± SD of three independent experiments. F, CNE2‐EV and CNE2‐NEK2 OE were injected subcutaneously into the left armpit of node mice respectively and tumor volumes were monitored every 3 days for 4 weeks. G, tumor weights were shown in these two groups. H, tumor volume assessments were shown in these two groups. I, Ki67 and NEK2 expressions were detected by IHC in the tumor engraftment from these two groups. CCK‐8, cell counting kit; IHC, immunohistochemistry; SD, standard deviation

To test the functional role of NEK2 in NPC, we overexpressed NEK2 by lentivirus mediated NEK2‐cDNA transfection in NPC cell line CNE2 and verified by qRT‐PCR and Western blot analysis on mRNA and protein levels (Figure 2A and 2 B). To examine the effect of NEK2 on NPC cell growth, CCK‐8 and colony formation assay were performed using NEK2 overexpressed CNE2 (CNE2‐NEK2 OE) and empty vector transfected CNE2 (CNE2‐EV). As a result, overexpression of NEK2 significantly increased cell proliferation in CNE2 compared with CNE2‐EV (Figure 2C; *P* < 0.05). Moreover, CNE2‐NEK2 OE showed a significant increase in colony formation compared with CNE2‐EV, indicating that high levels of NEK2 promote cancer cell proliferation (Figure 2D and 2E; CNE2‐EV 107 ± 10 vs CNE2‐NEK2 OE 286 ± 5, *P* < 0.05). We also tested the effect of NEK2 on NPC cell growth in vivo. In this study (five mice in each group), CNE2‐EV (8 × 10^5^) and CNE2‐NEK2 OE (8 × 10^5^) were injected subcutaneously into the left armpit of the node mice. Ten days after engraftment of the tumors, tumor formation was observed and tumor volume was monitored every 3 days. As shown in Figure 2F, overexpression of NEK2 promoted tumor growth in vivo (*P* = 0.03). To determine the correlation between NEK2 expression and cell proliferation, we performed Ki67 immunostaining on tumor engraftment. The cells were scored positive for NEK2 and Ki67 in the CNE2‐NEK2 OE compared with the CNE2‐EV group (Figure 2I: Ki67 score 5.3 ± 0.3 vs 2.7 ± 0.3, NEK2 score 5.3 ± 0.3 vs 2.3 ± 0.3), indicating that NEK2 promotes NPC cell proliferation in vitro and in vivo.

### 3.3 Knockdown of NEK2 by shRNA inhibits NPC cell growth

**Figure 3:**
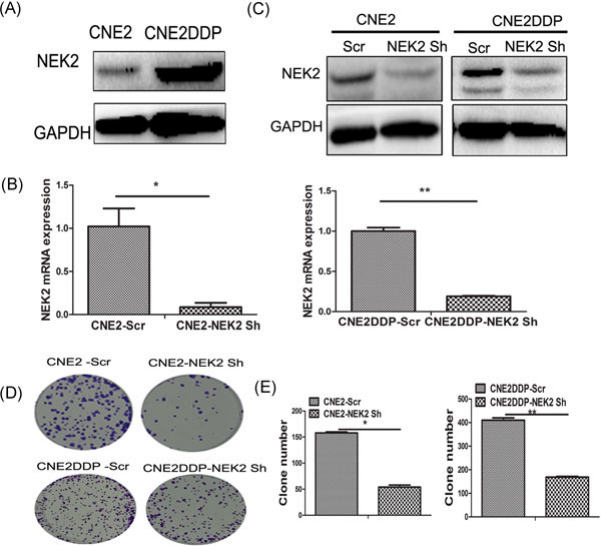
Knockdown of NEK2 by shRNA inhibits NPC cell growth. A, Western blot was performed to detect NEK2 protein expression in CNE2 cisplatin resistance cell (CNE2DDP) and CNE2. B, NEK2 mRNA expression in CNE2‐NEK2 Sh and CNE2DDP‐NEK2 Sh cell lines. C, NEK2 protein expression in CNE2–NEK2 Sh and CNE2DDP‐NEK2 Sh cell lines. D and E, the colony formations of CNE2‐Scr, CNE2‐NEK2 Sh, CNE2DDP‐Scr, and CNE2DDP‐NEK2 Sh were observed at day 10. Results were expressed as means ± SD of three independent experiments. GAPDH, glyceraldehyde phosphate dehydrogenase; mRNA, messenger RNA; NPC, nasopharyngeal carcinoma; shRNA, short‐hairpin RNA; SD, standard deviation

We found that NEK2 expression was significantly increased in CNE2DDP, a cisplatin resistance NPC cell line, compared with CNE2 by Western blot (Figure 3A). To further confirm whether NEK2 promotes NPC cell growth, target shRNA for NEK2 was used to knockdown NEK2 in CNE2 and CNE2DDP cell lines. RT‐PCR and Western blots analysis confirmed a remarkable downregulation of NEK2 expression in CNE2 and CNE2DDP cells after transfection of NEK2‐shRNA (Figures 3B and 3C).

Colony formation was performed to explore the effect of NEK2 knockdowning on cell growth in CNE2 and CNE2DDP. As shown in Figures 3D and 3E, downregulation of NEK2 induced significant inhibition of colony formation both in CNE2 and CNE2DDP (CNE2‐scramble 158 ± 2.8 vs CNE2‐NEK2 sh 54 ± 5.6, CNE2DDP‐scramble 410 ± 14.1 vs CNE2DDP‐NEK2 sh 169 ± 4.2).

### 3.4 NEK2 induces cisplatin‐based chemotherapy resistant in NPC

**Figure 4:**
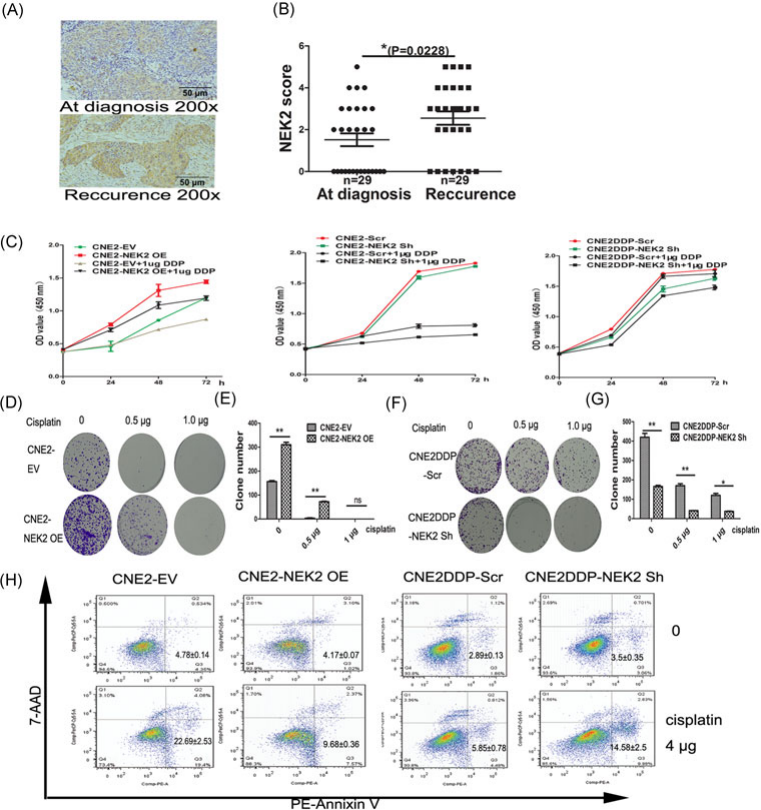
NEK2 induces cisplatin‐based chemotherapy resistant in NPC. A and B, NEK2 protein expression was detected by IHC in paired NPC sequential samples (*n* = 29). C, the proliferation of CNE2‐NEK2 OE, CNE2‐NEK2sh, and CNE2DDP‐NEK2 Sh was detected by CCK‐8 after cisplatin (1 μg) treatment of 24, 48, and 72 hours. D and E, the colony formations of CNE2‐EV and CNE2 ‐NEK2 OE treated with 0.5 or 1.0 μg of cisplatin. F and G, the colony formations of CNE2DDP‐Scr and CNE2DDP‐NEK2 Sh treated with different concentrations of cisplatin. H, apoptotic cells were detected by flow cytometry in CNE2‐EV, CNE2‐NEK2 OE, CNE2DDP‐Scr and CNE2DDP‐NEK2 Sh treated with 4 μg of cisplatin for 48 hours. CCK‐8, cell counting kit‐8; NPC, nasopharyngeal carcinoma

Since drug resistance is associated with poor prognosis, we detected NEK2 expression in 29 paired sequential NPC samples at diagnosis and recurrence. We found that NEK2 expression was significantly increased in recurrent samples (Figure 4A and 4B *P* = 0.0228). To guide the subsequent test, we have detected the half maximal inhibitory concentration (IC50) of anticancer drug cisplatin in CNE2 and CNE2DDP by CCK‐8 at the beginning of drug experiments. The IC50 of cisplatin for CNE2 and CNE2DDP is 1.74 ± 0.11 and 3.92 ± 0.10, respectively (Table 4). Furthermore, NEK2 overexpression significantly promoted cell proliferation and increased drug resistance to cisplatin in CNE2 compared with CNE2‐EV at 24 (*P* = 0.012), 48 (*P* = 0.004), and 72 hours (*P* = 0.001), knockdown of NEK2 significantly inhibited cell proliferation and increased drug sensitivity to cisplatin in CNE2‐Scr compared with CNE2‐NEK2 Sh at 24 (*P* = 0.010), 48 (*P* = 0.0271), 72 hours (*P* = 0.0277), the similar results in CNEDDP‐Scr compared with CNE2DDP‐NEK2 Sh at 24 (*P* = 0.0026), 48 (*P* = 0.0076), and 72 hours (*P* = 0.0137) (Figure 4C).

**Table 4:** Half maximal inhibitory concentration (IC50) of anticancer drug cisplatin in CNE2 and CNE2DDP cells

		IC50 of cisplatin, μg/mL
Cell line	Treatment	Mean ± SD^*^
CNE2	Cisplatin	1.74 ± 0.11
CNE2DDP	Cisplatin	3.92 ± 0.10^*^

Abbreviation: SD, standard deviation.

^*^ Results are presented as mean ± SD of three independent experiments, each done in triplicate.

To examine the effect of NEK2 on NPC drug resistance, a colony formation assay was performed using CNE2‐NEK2 OE, CNE2‐EV, CNE2DDP‐scr, and CNE2DDP‐NEK2 sh cells treated with different doses of cisplatin. As a result, CNE2 overexpressing NEK2 showed a decrease in their capacity to form colonies after cisplatin treatment at the concentration of 0.5 μg (310 ± 10 vs 72 ± 2) (Figure 4D and 4E), however, colony formation was almost completely inhibited in CNE2‐EV incubated with cisplatin at the same concentration (156 ± 4 vs 3.5 ± 1.5) (Figure 4D and 4E). Colony formation was not observed in both CNE2‐NEK2 OE and CNE2‐EV cells treated with high concentration of cisplatin (1 μg) (Figure 4D and 4E). Furthermore, CNE2DDP knock‐downing NEK2 showed a significant decrease in colony formation compared with CNE2DDP‐scr, indicating that NEK2 knock‐downing increased drug sensitivity to cisplatin in NPC cells (Figure 4F and 4G). To confirm whether NEK2 conferring drug resistance is associated with decreased apoptosis in NPC, the standard apoptosis assay was performed on the same two cell lines after treatment with the same drugs for 48 hours. Apoptotic cells were stained by the PE‐conjugated annexin‐V and determined by flow cytometry. As shown in Figure 4H, overexpression of NEK2 decreased cell apoptosis after addition of cisplatin compared with controls (9.68% ± 0.36 vs 22.69% ± 2.53), while knockdown of NEK2 increased cell apoptosis after addition of cisplatin compared with controls (percentage of apoptosis 14.58% ± 2.5 vs 5.85% ± 0.78).

## 4 DISCUSSION

NIMA related kinases, or NEKs, have discovered 11 distinct proteins called NEK1 to NEK11.^15^ NEK2, a member of the NIMA‐related family, has several putative roles in cell division, centrosome separation,^16^, ^17^ microtubule organization, centrosome integrity and dynamics,^18^ chromatin condensation,^19^ and spindle assembly physically.^20^, ^21^ On the pathologic function, aberrantly overexpressed NEK2 is associated with chromosome instability (amplification and misalignment), senescence, apoptosis, autophagy, metastasis, tumorigenesis, and drug resistance.^22^ NEK2 is relatively overexpressed in a number of human tumors. Evidence suggests that mRNA and protein levels are enhancive in tumor tissues or cell lines including breast cancer,^7^, ^21^ colorectal cancer^8^, ^23^, ^24^ neuroblastoma,^25^ as well as we previously reported in multiple myeloma,^11^ bladder cancer,^11^ glioblastoma,^11^ head and neck carcinoma, melanoma,^11^ hepatocellular carcinoma,^26^ and non‐small cell lung cancer and its subtype lung adenocarcinoma based on GEP databases.^27^ In this study, we further explored the status of the NEK2 in NPC, one of head and neck carcinoma, and confirmed that high NEK2 conferred poor survival, which indicated that NEK2 participated in the NPC pathogenesis.

The pathogenesis of NPC cannot be fully explained by the simple pathogenetic and develop fixed linear model because it may be a multistep and multipathway process as predicted by the tree‐like models for NPC carcinogenesis in which NPC can be classified into two groups, one marked by +12p11‐p12 as an early event, another one marked by +1q32‐q33 as a late event. Previously, we identified that BCAT1 located at +12p11‐p12 contributes to the early pathogenesis in NPC.^14^ Interestingly, in this study, we found that NEK2 is not only located at +1q32‐q33 but also had a great significance in the stage III‐IV and paired sequential recurrent patients with NPC indicating that NEK2 overexpression, as a late event, might be involved in the progression and recurrence of NPC.

Recurrence phenotypes may be acquired via therapy‐induced selection of resistant minor clones by direct adaptation to therapy of the original clone. NPC treatment strategies included radiotherapy plus induction chemotherapy (IC+RT), concurrent chemoradiotherapy only or plus induction chemotherapy in our study. We found that the patients with high NEK2 expression in the chemotherapy treatment have poor response to regimen. For the chemotherapy, cisplatin and other cisplatin‐based antitumor drugs have been widely administrated in several tumors including head and neck, lung, ovarian, and bladder cancers. One of the major clinical features of NPC is chemoradiotherapy resistance. The major anticancer mechanism of cisplatin‐based drugs is abnormal DNA damage repair and also several genes have been found to be associated with the cisplatin and other cisplatin‐based resistance of NPC, for example DNA repair gene RAD51L1,^28^ p53‐Mdm2,^29^ autophagy, and the epithelial‐mesenchymal yransition (EMT) process promote cisplatin resistance in NPC cells,^30^ BST2 confers cisplatin resistance via NF‐κB signaling in nasopharyngeal cancer,^31^ NEDD8 promotes tumorigenesis in NPC.^32^ Brain‐expressed X‐linked3, a CD271 receptor‐associated protein, contributes to cisplatin chemoresistance in NPC,^33^ TIMELESS confers cisplatin resistance in NPC by activating the Wnt/β‐catenin signaling pathway,^34^ MTA1 overexpression induces cisplatin resistance in NPC by promoting cancer stem cells properties,^35^ and Jab1/CSN5 contributes to radiation and chemoresistance in NPC through changing to the DNA damage and repair pathways.^36^ However, they cannot fully elucidate the mechanisms underlying NPC resistance. In this study, we indicated increased expression of NEK2 in patients with NPC with cisplatin‐based treatment. Moreover, we found NEK2 resulting in cisplatin resistance of NPC cells by performing CCK‐8 viability and colony formation.

Resistance to chemotherapy is a major problem for current cancer therapy. The mechanisms of resistance to chemotherapeutics share many features, such as alterations in the drug target,^37^ activation of the prosurvival pathways, proliferation, and activation of MDR protein as well as tumor initiating cells.^38^ Studies in multiple types of cancers have implicated the role of NEK2 in drug resistance. Except for the case where we reported that NEK2 induced bortezomib based drug resistance mainly through activation of efflux drug pumps, other groups indicated high NEK2 promotes paclitaxel and doxorubicin based drug resistance through inhibiting cell apoptosis in breast cancer cell.^39^ In this study, we firstly identified high NEK2 conferred to cisplatin‐based chemotherapy in clinical samples and also further confirmed the mechanisms of resistance to cisplatin through decreasing cell apoptosis.

In summary, for the first time, we demonstrated that expression of NEK2, which is located in the frequently amplified +1q32‐q33 region, increases at the advanced clinical stages (stage III‐IV) and paired recurrence of NPC. Increased expression of NEK2 protein could not be predicted for the poor survival but used as a novel biomarker for recurrence of NPC. In addition, high NEK2 confers to cisplatin‐based chemotherapy resistance in NPC samples. Downregulation of NEK2 by shRNA inhibited NPC cell growth and decreased drug resistance in vitro. Thus, targeting NEK2 has the potential to eradicate cisplatin‐based chemotherapy‐resistant NPC cells.

## FUNDING INFORMATION

This work was supported by the National Natural Science Foundation of China (81570205, 81400169, 81630007, 81372835, 81670143), Strategic Priority Research Program of Central South University (ZLXD2017004), Hunan Province Natural Science Foundation of China (2015JJ2158), SKLEH‐Pilot Research Grant (ZK16‐04), Fundamental research funds for the Central Universities of Central South University (2018zzts848), Open sharing fund for valuable instruments and equipment for the Central Universities of Central South University (CSUZC201833).
